# Suitable Sweden: Co-producing Sweden Through Reproduction, Technological Development and International Aid in the Mid-twentieth Century

**DOI:** 10.1007/s10912-025-09945-6

**Published:** 2025-05-01

**Authors:** Morag Ramsey

**Affiliations:** Department of the History of Science and Ideas, Uppsala University, Engelska Parken, Thunbergsvägen 3P, Box 629, 751 26 Uppsala, Sweden

**Keywords:** Sweden, Reproductive research, Birth control technologies, Overpopulation

## Abstract

In the mid-twentieth century, there were worries about overpopulation globally. Birth control — an often-sensitive topic — was made to be an issue of urgency through an overpopulation discourse. It stopped being associated mainly with sex and started to be associated with ending world starvation. Some nations were seen as requiring help; some nations were seen as politically sensitive to birth control; some nations were seen as financially capable; and other nations, such as Sweden, were seen as well suited for developing birth control technologies. This paper traces how Sweden became a main global protagonist in international aid that focused on reproduction through local birth control development. By using perspectives from science and technology studies, I examine the specific networks and actors that made birth control development and testing possible in Sweden in the mid-twentieth century. Centering around the testing of intrauterine devices and abortion pills in the 1960s, this paper shows how technological development in Sweden co-produced understandings of reproduction and the conditions for reproductive research infrastructure. It argues that the technological development of birth control is an important and yet understudied aspect of how Sweden situated itself internationally when it came to the overpopulation scare.

## Introduction

In late 1968, an influential figure at an American private grant foundation speculated that Sweden would be a good choice as a reproductive research site. In his communications with a Swedish researcher, he had learned that investigations “can proceed much more rapidly to clinical trials on human patients under Swedish medical regulations than can their opposite numbers in the United States under the more conservative FDA regulations” (Confidential Letter 1968). In this era, and in the specific area of reproductive research, speed was seen as a necessity. Since the early 1950s, several countries had struggled to make population growth, family planning, and subsequently sex, matters of urgency on a global scale (Connelly 2008). Now, in the 1960s, there was a consensus in powerful global institutions that population growth was a serious issue that needed immediate solutions (Bashford 2013). In Sweden domestically, there was no perception of an overpopulation issue, but, as I will show, there was interest in contributing solutions to this global problem.

Curbing population growth was not seen as straightforward. The initial backlash in the 1950s against the topic was due to the private, controversial status of sex. Attempts in the early 1950s by the World Health Organization (WHO) to include birth control programs were met with hostility from many countries. These countries compared birth control to means of suicide, describing it as going against nature and as morally wrong. There were threats to boycott the WHO if the organization took on a broad family planning role and rejection of the WHO’s role in population control measures, as many critics did not consider population control to be a medical issue (Connelly 2008). Sweden was one of the first countries to include birth control in its foreign aid when it provided family planning assistance to Ceylon in 1958, but not many other countries engaged in such aid projects (Berg 2010). By the late 1960s; however, a reproductive research hub was desired by many actors. Domestic legislation still made it difficult for some countries, like the USA, to address reproductive topics. Abortion was a contentious issue, and domestic legislation concerning abortion prohibited some forms of related research (Oudshoorn 2003). But Sweden was an alternative option. How, exactly, did Sweden come to play a central role in international efforts to curb overpopulation growth?

Using theoretical tools from science and technology studies (STS), this article traces some of the first clinical trials of reproductive technologies in Sweden, showing how research that began in the early 1960s created conditions for international family planning and reproductive research collaboration. Focusing on critical moments in the testing of intrauterine devices (IUDs) and abortion pills shows how technological development had a significant impact on conditions for international research collaboration. By using the concept of co-production, I show how various actors came together, influenced one another, and made it possible for Sweden to assume this role.

For the concept of co-production, I rely on the work of Jasanoff (2004), which emphasizes how social and natural orders are produced together, shifting the emphasis from the impact of single actors or technologies and instead examining innovation as a collaborative, messy, and ongoing process involving multiple actors. Here, the concept of co-production is mainly used to emphasize how ideas about *Sweden* were formulated in these reproductive research and aid networks. Creating and testing the two abovementioned types of birth control were collaborative and messy efforts that overlapped with foreign aid concerns. By examining how the different actors in these networks conceived of Sweden, we can see how the opportunities for science were intimately connected to how the nation was imagined and, in turn, to how Sweden was made to practically and physically support these imaginations.

Centering Sweden in the historiography of overpopulation and birth control development shows how specific Swedish historical conditions and actors contributed to international efforts to curb overpopulation. While Sweden is often considered to be a progressive and sexually liberated country, closely examining how the country became an important global partner in birth control development complicates any easy explanations as to why. As will be shown, specific dynamics of Swedish state institutions, research culture, international collaborations, and global interests played important roles in making Sweden an appealing reproductive research hub. This paper argues that small-scale clinical trials helped secure Sweden’s position in development aid internationally during the mid-twentieth century. The research also contributes to scholarship on the Nordic model and the way particular ideas of Sweden were formulated and circulated and to what effect (Byrkjeflot et al., 2022). Previous scholarship has shown that science and technology were important components of building the mythology of Sweden’s unique form of governance and politics during the mid-century period (Lundin and Stenlås, 2010). As shown here, technology, science, and expertise constitute an important framework for understanding Sweden’s decisions and policy on sex and birth control.

To answer the question of how Sweden came to have a central role in international efforts to curb overpopulation in the mid-twentieth century, I used: historical material from clinical trials; correspondence among researchers, government officials, and non-profit-sector workers; parliamentary minutes; WHO records; plans to develop a reproductive research hub; and media material. This selection of material allowed an examination of key actors and of how they engaged and influenced one another, highlighting the importance of ascribing various qualities to Sweden.

The article is divided into four sections. The first introduces previous research, situating the current paper within existing fields of scholarship. The second section explores the development of two birth control technologies, showing how work on the IUD and the abortion pill created favorable conditions for reproductive research. The third section focuses on international efforts to create a reproductive research center and Sweden’s important role in the related discussions. The fourth section shows how these actors, who focused on reproductive research, foreign aid, and overpopulation, co-produced specific versions of Sweden.

## International development aid, reproductive research, and the Nordic model

Previous research has thoroughly covered the overpopulation scare of the mid-twentieth century (Bashford 2013; Connelly 2008; Dowbiggin 2008). Scholars have identified intricate networks of actors organizing to curb population growth, highlighting the diversity of those preoccupied with population—ranging from eugenicists to environmentalists—and illustrating how birth control became a mainstream topic in international forums such as the United Nations (Bashford 2013). A focus on food, starvation, and planetary limits has been well documented in international networks focused on overpopulation. While Sweden is occasionally mentioned in these global histories, the main focus has not been on Scandinavian countries. However, the global overpopulation scare gave rise to Swedish participation in international development aid in which family planning played a key role.

Regarding the Swedish emphasis on family planning, various scholars have examined the emergence of Swedish international aid, including the establishment of the Council for International Development (NIB) in 1962, which then became the Swedish International Development Cooperation Agency (SIDA) in 1965 (Berg et al. 2021). Berg has shown continuity between Sweden’s domestic interwar population policy and the country’s international population control efforts in India (Berg 2010). The impact of overpopulation worries on international collaboration and family planning efforts has been studied at the institutional and policy levels, including in analyses of key historical figures.

Scholars have also shown that the role of development aid has been important to Nordic countries more broadly, as these countries have considered aid a moral obligation and were often generous donors (Diurlin 2022; Engh 2022). Nordic countries have historically pushed for the inclusion of new areas in aid, whether that be new recipient countries, new areas of interest, or new target groups (Engh 2022). In the late 1950s, for example, Ceylon was seen as a suitable recipient country for aid to counter overpopulation, and family planning was a new area of interest. In Sweden in the 1960s, the Social Democratic party also used development aid targets to appeal to younger voters, whom the party perceived as more engaged with international issues (Engh 2022). As previous scholarship has illustrated, international aid has played an important role in the Swedish welfare state and has been of special interest to Nordic countries (Diurlin 2022; Engh 2022). Investing in aid has been seen as an effective form of soft power, allowing the Nordic countries to engage on an international stage as “moral great powers” (Diurlin 2022).

Recently, there has also been increased interest in reproductive research conducted in Sweden, especially in the mid- to late twentieth century. The conditions for such research, key actors and institutions, and the impact of reproductive research have begun to be analyzed, identifying a tight network of actors and a range of consequences springing from these research endeavors (Franzén 2022; Jülich 2018a; Ramsey 2021; Tinnerholm Ljungberg 2021). Importantly, the intersection between Sweden’s abortion culture and laws and the opportunities for reproductive research has been investigated, showing that fetal research, for example, had already begun in the 1950s in Sweden, using remains from legal abortions (Jülich 2018b). Scholars have also analyzed the underlying complexity of various reforms and legislation that have gradually liberalized access to contraceptives and abortion services in Sweden, showing how an amalgamation of government authorities, individuals, laws, medical professionals, and student movements contributed to significant changes from the 1930s forward (Lennerhed 2017a, 2017b; Palmblad 2000; Tydén 2010). Motivations for providing birth control have ranged from safeguarding against venereal disease, promoting eugenic ideals, empowering individuals, and fortifying medical services (Lennerhed 2017a; Tydén 2010). A single progressive ideal of individual autonomy and shame-free sexuality has not been the prevailing motivator over the decades. Instead, scholars have shown that managing reproduction was seen as a component of a successful welfare state and that the underlying motivations for legal and policy changes reflected this philosophy (Palmblad 2000). The intricacies of the Swedish welfare state’s reproductive management over the twentieth century have been an important factor in creating opportunities for reproductive researchers.

Controlling reproduction at a population level has also been of interest to scholars. While some studies have focused on the benefits of welfare policies for the population, such as pursuing gender equality goals with the establishment of parental leave in 1974, other scholars have focused on the more nefarious efforts of the eugenics movement (Nyberg 2012; Tydén 2010). Between 1930 and 1950, around 35,000 people were sterilized for eugenic and/or social reasons by the Swedish state. Most of those sterilized were women, and Tydén (2010) argued that these sterilizations were in practice compulsory even though they were considered elective at the time. The complexities of how eugenics was used in the Swedish democratic setting and by governments that were seeking to improve the lives of and protect poor and marginalized groups have been helpfully analyzed elsewhere (Roll-Hansen and Broberg 2005; Tydén 2010). What is remarkable for this study is the shared goal of curbing the birth of undesirable/undesired children via eugenic sterilizations for domestic populations and, as will be illustrated, family planning initiatives abroad.

There is also a significant field of study of the “Nordic model,” a term referring to similarities among the Nordic countries, especially connected to their political practices. The idea that the Nordic countries share overarching qualities, often connected to the establishment of progressive policies, the expansion of the welfare state, and the promotion of democracy, has been shown to have influenced the image and understandings of the countries both internally and externally (Diurlin 2022; Engh 2022). In Sweden’s case, early on the country earned its own international taglines connected to the Nordic model, referring to a “middle way” and “the Swedish model.” In the 1930s, Sweden encapsulated the idea of a “middle way” between Soviet socialism and American capitalism (Hale 2003). Since then, what has been considered “the Swedish model,” such as welfare state-initiated housing reforms, economic policies, and healthcare programs, has been promoted abroad by Swedish institutions, diplomats, and intellectuals (Andersson 2009; Byrkjeflot et al. 2022). Previous research has followed the concentrated efforts of Swedish institutions to relay these specific and positive images abroad (Glover 2012). In this sense, qualities that contributed to Sweden’s inclusion in the Nordic model were also used to depict Sweden as unique and special abroad.

While the idea of the Nordic model offers an analytical framework, scholars have also cautioned that leaning on the term can conceal variation among the countries. In terms of the Nordic model and development aid, Engh (2022) has argued that the concept of “the Nordic model” can mask differences in policy, outreach, and practice among Nordic countries and differences among time periods. In a recent anthology, Byrkjeflot et al. (2022, 2) defined the Nordic model along the lines of a collective singular, as “a concept that is mentioned in singular, but in praxis is used in different ways in space and over time.” In this article, I join discussions of the effects of positive Swedish branding internationally during the mid-twentieth century, which can be related to ideas of the Nordic model more broadly. In particular, I emphasize the deep and meaningful relationship between reproductive research and development aid and how positive ideas of Sweden were co-produced by actors in these professional fields. Recent scholarship has shown that there is still work to be done in understanding exactly how ideas of the Nordic model circulated (Norén et al. 2023). The present article highlights how small-scale, local reproductive research practices were part of this myth-making during what is considered the classic Nordic welfare epoch.

This paper builds on these intersecting fields of scholarship on overpopulation, international aid, reproductive research, and the Nordic model. I demonstrate how foreign aid and reproductive research were deeply intertwined, highlighting the importance of technological development and the use of specific birth control methods and how this affected legislation, discourses about sex, and opportunities for international collaboration. Linking local clinical and laboratory practices with larger sociocultural concerns also illustrates how conditions for reproductive research were co-produced by actors at different levels. In her own work on the WHO and population control, Oudshoorn (1997, 41) argued that analyzing intermediary organizations, such as the WHO, “enables us to go beyond a too-narrow focus on the micro-sociological dynamics of laboratory work, to include the macro and meso-sociological dimensions of science and technology.” Here, I couple an analysis of local clinical trials with “concerns that go beyond the laboratory,” identifying both specific ways actors in Sweden were influenced by international affairs and how Swedish variables in turn contributed to international research infrastructure (Oudshoorn 1997, 41). The technological development of birth control is an important and yet understudied aspect of how Sweden situated itself internationally when it came to the overpopulation scare. This further contributes to discussions of depictions of Sweden abroad, as the technological development of birth control created new ways of positioning Sweden relative to outsiders, and was also important to how outsiders then perceived and understood Sweden.

## Developing birth control

In the 1960s, Sweden, much like other countries, saw the introduction of new birth control technologies. The contraceptive pill, the IUD, and new abortive technologies were all introduced (Ramsey 2021). While some of these birth control methods were imported from abroad, others were being developed and tested in Sweden. Various reproductive management initiatives in the decades prior had helped to create favorable clinical testing conditions for Swedish researchers.

The 1930s had been an active decade for reproductive management by both state and private actors. The Social Democrats had been elected in 1932 and, over the following years, oversaw various social welfare policies, many of which were reproduction related (Björklund 2012). This decade saw official worries about depopulation, with a special commission set up to investigate the issue (Lennerhed 2009). The Population Commission was active from 1935 to 1938, publishing the report *The Sexual Issue*, in which it stressed the importance of sex education in school and unbiased discussions of sexuality, and recommended repealing the 1911 Contraceptive Act, which had banned spreading information about contraceptives (Lennerhed 2000). Part of the Commission’s assessment linked a declining population to tensions arising from gender inequality and industrialization (Lennerhed 2009). At the same time, a sterilization law also came into effect in 1935, allowing sterilization without consent as a measure to improve the “quality” of the population by targeting people considered intellectually disabled (Broberg and Roll-Hansen 2005). Meanwhile, the Swedish Association for Sexuality Education (RFSU), an organization founded in 1933 by Elise Ottesen-Jensen, opened clinics, advocated legalizing contraceptives and abortion, and offered sex education (Lennerhed 2002). In 1938, the Abortion Act was also passed as a response to the criminal code, which had criminalized abortion. The Abortion Act legalized three types of abortion, i.e., those done for medical, eugenic, and humanitarian reasons (Lennerhed 2017a, b). This meant that if a woman’s health was seen to be harmed by her pregnancy, if she suffered from hereditary illnesses, if she had been raped, or if the pregnancy was the result of incest, abortion was permitted.

During this decade, the Swedish state grappled with various ways of managing reproduction and population. Legal and policy changes liberalized access to abortion and contraceptives (although there were still restrictions), while there were also initiatives to sterilize people. RFSU worked to spread sex education and offer clinical services, while the Population Commission also recommended various measures to change society’s relationship to sex. There were clear tensions in managing reproduction, which entailed improving the lives of some people while restricting the reproductive choices of others. Notably, legalizing the spread of information on contraceptives and, very importantly, legalizing certain types of abortion made conditions for reproductive research more favorable in the following decades. As other scholars have shown, fears of global overpopulation helped to make birth control development a matter of urgency (Oudshoorn 1997; Takeshita 2012). Simple reproductive technologies that could be used by many people were the dream scenario for actors involved in family planning (Takeshita 2012).

### Testing IUDs

In the early 1960s, there was increased interest in testing and using IUDs in Sweden. A 1938 royal proclamation had banned the use of IUDs, classifying them as abortifacients in an attempt to reduce illegal abortions done by mechanically inserting objects into the uterus (Royal Proclamation 1938). This meant that for several decades Swedish physicians did not consider the IUD a meaningful type of birth control, although the technology was being used in other countries (Dugdale 2000). But by the late 1950s, new reports on clinical trials of IUDs sounded encouraging to several Swedish physicians (Preliminary Report 1964). They turned to the National Board of Medicine for permission to test new types of IUDs that had been developed abroad. The National Board of Medicine was a government authority under the Ministry of Health and Social Affairs that oversaw various health services in Sweden. As IUDs were banned and considered abortifacients, there would need to be special consideration of their use in any kind of trial. Starting in the early 1960s, Sweden had been providing IUDs in its family planning projects abroad in Ceylon and in Pakistan, another country seen to have excessive population growth (Berg et al. 2021). This would later create tension around IUD use, as the reproductive device was included in foreign family planning efforts but was still not available to Swedish women.

The IUD was a birth control technology that had been invented in the early 1900s (Dugdale 2000). While inserting an object into the cervix to prevent pregnancy was not a new idea, IUDs were designed to be compressed and then inserted through the cervix into the uterus. The Gräfenberg ring, an IUD made out of silk thread and silver wire, was one of the more notable IUD designs of the early to mid-twentieth century (Rusterholz 2017). By the early 1960s, two new IUDs were attracting international attention, one IUD designed by Lazar Margulies and another by Jack Lippes (Takeshita 2012). The Margulies spiral and the Lippes loop (named after their inventors and their respective shapes) were made out of flexible plastic and designed to be straightened, inserted into a long tube applicator that was inserted into the uterus, and then pushed through an applicator tube; once the IUD had passed through the tube and entered the uterine cavity, it would spring back into shape. This permitted the insertion of these IUDs to be less painful than the insertion of earlier models (Connelly 2008). While researchers were modifying and trying different shapes, exactly how IUDs prevented pregnancy was not well understood. The classification of IUDs as abortifacients was then open to debate.

In 1963, several physicians employed at RFSU and the Mental Health Bureau (MHB) in Stockholm petitioned the National Board of Medicine to allow testing of the new IUDs (Preliminary Plan 1963). In their proposal, the researchers informed the board of practical measures that they would take to avoid any abortifacient effect of using IUDs (Preliminary Plan 1963). After selecting trial participants, the IUD would only be inserted during the woman’s menstrual period, ensuring that there was no pregnancy to disturb. This was an important detail, as the Abortion Act of 1938 was still in effect, and an abortion could only be legally secured through rigorous screening (Ramsey 2021). To be eligible for an abortion, a woman would need to meet several medical professionals and a social worker over the course of a few weeks, adding time and labor to any clinical testing design. As will be illustrated, the Abortion Act both created opportunities for and slowed down clinical testing. In the case of the IUD, researchers were careful to position their clinical trials as testing a contraceptive, *not* an abortifacient, simplifying the trial process.

Starting in January 1964, physicians at RFSU and MHB began testing IUDs with official permission from the National Board of Medicine (To Acting Physicians 1966). The men involved in these trials were well connected in international forums such as the International Planned Parenthood Federation (IPPF) and the Population Council in New York, and through their connections were able to acquire the Margulies spiral (Preliminary Report 1964). The Swedish IUD work would also be included in a larger international study run by the Population Council. A five-year clinical trial project called the Cooperative Statistical Program (CSP) had been launched in the USA to create broad statistical data on IUD use (Takeshita 2012). IUDs were considered to be well suited for family planning projects as they were seen to require only very passive participation from recipients. It was a birth control method that family planning actors imagined could simply be inserted in women and left there for long periods of time, proving less fallible than other methods that required more active engagement, such as the contraceptive pill (Dugdale 2000). However, the IUD had acquired a poor reputation in the USA, and with CSP there was hope that gathering enough data would rehabilitate the device’s image (Takeshita 2012). While most of the testing sites were in the USA, there were three sites abroad, including one in Stockholm.

After several years of testing IUDs, the Swedish physicians submitted a report to the National Board of Medicine. Overall, the researchers were satisfied with their trial. They had inserted IUDs into roughly 250 women and had found the results promising (To Acting Physicians 1966). However, testing IUDs was not the same as making this means of birth control widely available, and researchers involved in testing the devices were also pushing to change the law. In April 1965, one IUD researcher and a Stockholm-based lawyer wrote to the Department of Justice, asking that the Royal Proclamation of 1938 banning IUD use be repealed (The Working Committee 1965). In their letter, they argued that the IPPF had recommended the use of IUDs and that ongoing scientific investigations of IUDs in Sweden showed promise. They wrote that these studies had “not provided any evidence whatsoever that these intrauterine devices would act as an abortifacient” (The Working Committee 1965). They added that IUDs were simply an alternative contraceptive option for many women, especially women who could not rely on other methods. Through clinical testing, IUDs were transitioning from a banned abortifacient to a useful and safe contraceptive option.

After conferring with their scientific advisors, the National Board of Medicine took the issue of IUDs to the Ministry of Health and Social Affairs (Sundberg to the Ministry of Health and Social Affairs 1965). Two memos by well-respected Swedish physicians were also sent to the head of the Ministry of Health and Social Affairs, explaining the status and use of the IUD both in Sweden and abroad (Tottie 1965). In late 1965, the National Board of Medicine assessed the status of the IUD and, after weighing the evidence presented, suggested that the provision on IUDs in the Royal Proclamation of 1938 be removed (To the King 1965). In February 1966, the decision was officially taken, coming into effect in May 1966. While IUDs were removed from the list of abortifacients, only IUDs that had been approved by the National Board of Medicine would be permitted for use in Sweden.

Sweden had been providing IUDs through its foreign family planning initiatives since the early 1960s, and by the summer of 1966, IUDs were available at select clinics as a birth control option for Swedish women. International pressures to curb overpopulation had propelled the IUD into various testing circumstances, including local Swedish sites. However, as will be outlined below, changing the IUD’s status from abortive to contraceptive would not go unchallenged. Discussions of reproductive definitions, such as fetus, abortion, contraception, anti-development, and anti-conception, would occur in debates over what was right and legal when it came to birth control use and testing. The overlapping work on abortion pills would only add to the confusion.

### Developing abortion pills

While IUDs were being tested at RFSU and MHB, another researcher affiliated with both institutions would submit an application to the State Pharmaceutical Laboratory, an institution under the National Board of Medicine, to clinically test abortion pills. In November 1965, Lars Engström applied to conduct clinical trials of a new abortion pill: F6103 (Application 1965). Unlike the testing of IUDs, Engström explicitly sought to test an abortifacient and would therefore need to run his trials in accordance with the Abortion Act of 1938. This required screening and selecting trial participants based on their legal status of being eligible for an abortion, a process that took time and resulted in longer pregnancies than otherwise.

For the first round of testing, Engström abided by these legal terms, waiting for potential trial participants to meet with gynecologists, physicians, and social workers and to be granted legal permission for abortion (Bacic et al. 1970). However, Engström wanted to develop an abortion technology for use in early pregnancy, and the process of assessing women for legal abortion was slowing down their inclusion in his trial. In April 1966, Engström submitted a new trial application in which he hoped to give F6103 to women who had refrained from using contraceptives and then had missed their menstrual period (Application 1966). This would allow Engström to test F6103 as early as possible in a pregnancy, differing from his first clinical trials. However, he needed advice from the National Board of Medicine as to how to proceed legally.

The National Board of Medicine employed a lawyer, Hans Thornstedt, to look into the issue of clinically testing F6103. Was it legally possible to test abortifacients in the manner Engström laid out? While the Abortion Act complicated the trials, the legal status of abortion also provided incentives for the Swedish state to develop safe and efficient abortion technologies, as it was responsible for providing this medical service. Over the summer of 1966, Thornstedt investigated the legal precarity of Engström’s proposed research. The Swedish criminal code, which initially saw anti-abortion measures added in 1734, explicitly focused on fetal displacement. Accordingly, much of Thornstedt’s (1970) analysis concerned the modern definition of a fetus. Two main legal definitions of fetuses were in use, one defining a fetus as a fertilized egg and the other as a fertilized egg implanted in the uterus (Thornstedt’s Letter 1966). Thornstedt found more support in the legal literature for the first definition, which stated that a fetus was a fertilized egg. This meant that a fetus appeared even earlier in pregnancy, so administrating F6103 to women in very early pregnancy would still be displacing a fetus, and as such the trials of F6103 were seen to be in conflict with the criminal code.

Thornstedt’s analysis was fairly clear: the criminal code did not leave room for scientific experimentation. However, the lawyer still had a suggestion: create a legal amendment in order to be able to conduct clinical trials on pregnant women. The National Board of Medicine agreed with this recommendation and in October 1966 submitted a proposition to the Swedish parliament (Proposition 18, 1967). The proposition included two referrals, one from the prosecutor general and another from the 1965 Abortion Committee. The Abortion Committee had been set up by the state to investigate abortion conditions in Sweden and to eventually make recommendations about existing laws and practices. In the referral approving a legal amendment, the Abortion Committee wrote:

An agent whose use would cause early pregnancy to be discontinued without surgery, must be seen as a major advancement. What seems to be the most significant thing, however, is the importance of such an agent for family planning. In all endeavors to assist developing countries, one of the more important elements must be to help master the overpopulation problem. Although this problem is not relevant to our country, it is internationally so significant that Sweden too should help in approaching its solution by making an easy and effective means of birth control available. (Proposition 18, 1967, 5)

Alongside state incentives to create technologies to make abortion easier and safer to administer to Swedes, there was the ever-present incentive of addressing global overpopulation. In the mid-1960s, discussions of overpopulation were presented in Swedish media, with major newspapers covering the issue. The connection between overpopulation and starvation was prevalent, just as it was in international coverage (Bashford 2013). Reproduction researchers, such as Engström, had also actively participated in public discussions of how Sweden could help fight global overpopulation (Schoug 1966). By 1966, newspaper articles were outlining how Sweden could prevent millions from starving by providing family planning assistance and through contraceptive research (Svenska Dagbladet 1966a, 1966b). Sweden’s role in fighting global overpopulation was being co-produced in public, government, and private spaces as intimately connected to family planning and birth control research.

This emphasis on overpopulation brought together parliamentarians who were otherwise worried about the implications of allowing clinical testing on pregnant women. After submitting the proposition, the Swedish parliament then discussed the merits and potential disadvantages of this legal change in the spring of 1967 (Ramsey 2021). While majority were in favor, those with objections wrote of their concerns in various motions circulated to their colleagues. The discussions in parliament that followed made it clear that uncertainty around reproductive concepts was causing confusion over what was occurring in these clinical trials. While Thornstedt had relied on legal interpretations to assess the trials, other medical experts would have disputed his definition of fetus. There was also uncertainty as to when F6103 worked and how, making it difficult to know whether it disrupted an existing pregnancy or prevented one from occurring (Ramsey 2021). In parliament there were disputes over what a fetus was, how F6103 might actually work, and the differences in biological phases in individual women. Some parliamentarians were concerned about “defenseless life,” some over the clinical trial conditions, and others over the ambiguity from lawyers and researchers over what was actually occurring in the trials (Parliament Protocol 1967). While some parliamentarians opposed the research or worried about its repercussions, most of them still conceded that fighting global overpopulation was warranted. This macro-social political concern was a key influence in creating local clinical trial opportunities. In their interactions, reproductive researchers, parliamentarians, government workers, and media outlets were co-producing Sweden as a country engaged with the important moral question of global overpopulation. While there were many different aspects to discuss when it came to reproductive research, and in some instances disagreement over what should be done, many of these actors emphasized that Sweden needed to engage with the issue of global overpopulation. In this way, Sweden was being co-produced as a country concerned with global overpopulation. This concern, however, as the reproductive research exemplified, was being curbed by domestic legislation.

One issue raised by Ingrid Segerstedt Wiberg in parliament connected the legal status of the IUD to the ongoing abortion pill puzzle. In this way, the decision taken the year before to legally allow IUDs was brought into the discussion of abortion pills, further confusing the issue of the technology’s legality while also acting as a form of precedent.

### Clinical practices, the law, and favorable reproductive research conditions

While it was a relatively smooth process to adjust the legal conditions for IUD use, it did not go undisputed. In its role as a government authority, the National Board of Medicine had advised amending the royal proclamation and the government had agreed. The Swedish parliament, however, was not involved in the decision, which, as it would turn out, created issues. A parliamentary committee called the Committee on the Constitution examined the government’s decision and found that “use of plastic intrauterine devices could come into conflict with the Criminal Code’s provision on abortion. With this in mind, the parliament should have participated in the device’s legislation” (Committee’s Decision 1967).

The Committee on the Constitution’s assessment made ripples in the Swedish press, as newspapers wrote about the legal tensions of making IUDs available (Michanek 1967). This occurred in the spring of 1967, when the parliament was meeting to discuss the proposal to allow clinical trials on pregnant women. Some parliamentarians, such as Segerstedt Wiberg, thought that the government’s decision to permit IUDs had been taken erroneously and was in breach of the criminal code, while others did not see an issue with it, emphasizing that the decision had been made based on information from the top authority on medical issues in the country—the National Board of Medicine (Parliament Protocol 1967).

In taking a decision on IUDs, the National Board of Medicine had consulted researchers and physicians involved in IUD research in Sweden and had grappled with the boundary between anti-conception (preventing pregnancy) and anti-development (ending a pregnancy) technologies. As noted, researchers were very clear that inserting an IUD during a woman’s menstrual cycle prevented the device from acting as an abortifacient, which ultimately helped to alter this birth control device’s status from that of abortive to contraceptive. In the case of abortion pills, researchers were actively interested in using reproductive technologies on pregnant women. So, while discussions of IUDs shifted the device to being a contraceptive, the issue of testing F6103 explicitly required consideration of whether pregnant women could be trial participants.

Both the IUD and abortion pill parliamentary debates were covered by major Swedish press outlets. An article in the major Swedish evening newspaper *Expressen* provided dramatic coverage of the IUD debacle brought forward by the Committee on the Constitution. In this piece, the journalist wrote that a member of parliament had run from the Committee on the Constitution’s meeting in order to warn the head of the Ministry of Health and Social Affairs and the head of the Justice Department that the decision the government had taken on IUDs the year before was to be formally criticized (Michanek 1967). In this article, the journalist speculated that the Minister for Foreign Affairs had pushed for the IUD’s change in status from abortive to contraceptive, writing, “She did not want to risk having those in Pakistan say: In Sweden you did not approve the IUD, but you want to insert them in Pakistani women. Are you not ashamed?” (Michanek 1967).

The potential hypocrisy of providing IUDs abroad while not allowing them to be used in Sweden was explicitly brought into public discussion of reproductive technology. In this discussion, providing technologies to fight starvation by reducing the global population was only a noble aspiration if the same means could be used domestically. Changing the legal status of the IUD was then seen as necessary for participating in the endeavor to supply family planning abroad. It also initiated discussions in various government authorities over birth control technologies, what they were intended for, and how to support their development. Creating legal support to test reproductive technologies on pregnant women then fell into a wider and established view of family planning, birth control technologies, and Sweden’s role abroad.

While there was discord over the IUD decision, the reproductive technology did not lose its new legal status. In the spring of 1967, proposition 18 was passed into the *Law of clinical trials of certain means of birth control*, coming into effect 6 June 1967 (Law 1967). The micro-clinical trial practices in Sweden, occurring on a small scale and involving a limited network of researchers, had very real legal ramifications for potential birth control practices in Sweden, for future reproductive research, and, as will be illustrated, for international collaboration.

## Fighting starvation through reproductive research

There was a relatively tight network of individuals working on reproductive research in Sweden in the 1960s. RFSU and MHB were important institutions that allowed experimental medicine and clinical trials, and often the researchers involved in testing IUDs and abortion pills performed their work in collaboration with these two sites. Individuals involved in more regulatory aspects of the clinical work, such as expert advisors at the National Board of Medicine, were also, often, conducting reproductive research of their own. Engström, who headed the abortion pill work on F6103, was one of the two physicians who wrote a lengthy memo to the head of the Ministry of Health and Social Affairs when the legal status of the IUD was about to be assessed (Engström to Social Department 1966). Later in the 1960s, other researchers working on different abortion pill developments would also be employed by RFSU and simultaneously work to expand the use of IUDs in Sweden (Ramsey 2021). Reproductive research and abortive and contraceptive clinical initiatives occurred in a close network of actors.

Alongside these clinical research-oriented spaces, there was another relevant field: family planning and foreign aid. Various individuals with reproductive research and medical backgrounds also involved themselves in family planning efforts. Examining the intersection of reproductive research and foreign aid shows how these two areas came together, as Swedish media proclaimed they should, to fight global overpopulation. It also demonstrates how the micro-clinical practices of developing new birth control methods in Sweden influenced conditions for international collaboration.

### Creating a research and training center

While family planning and reproductive research had been difficult topics to integrate into large international forums, by the 1960s the mood was changing (Bashford 2018; Weisz and Olszynko-Gryn 2010). By then, the United Nations, through its specialized agency the WHO, had entered into the arena of family planning and fertility control (Eighteenth World Health Assembly 1965). Improving knowledge of reproductive processes was seen as important for tackling overpopulation issues, and several influential actors in family planning would be brought together in an effort to establish reproductive research infrastructure (Harkavy and Maier 1979). While key American individuals and organizations were interested in setting up a research center, initially Swedish actors played important roles.

In the late 1960s, representatives from the American private organization the Ford Foundation, from the Karolinska Institute in Stockholm, and from the Swedish International Development Cooperation Agency (SIDA) began making plans to establish a reproductive research center. The Karolinska Institute had hosted research on hormones and reproduction for decades, SIDA had experience in offering family planning services in its foreign aid programs, and the Ford Foundation had been investing in both family planning and reproductive research since the 1950s (Berg 2010; Harkavy et al. 1968). These actors linked practical knowledge of supporting reproductive research, the intricacies of family planning, and the particularities of funding. In this era, the existing family planning methods were seen by some as inadequate and as not curbing population growth quickly enough (Johannisson 1971). Expanding knowledge of basic reproductive processes could potentially lead to new strategies.

Creating what was considered a “Manhattan Project” for reproductive science was appealing to several actors concerned with overpopulation, family planning, and birth control (Population Memoranda n.d.). Oscar Harkavy, Ford Foundation Program Officer at the Population Office, wanted to improve reproductive knowledge by having a centralized administration with funding that could support several research centers globally (Population Memoranda n.d.). In this endeavor, international collaboration was seen as essential. While there was much more willingness to engage with family planning and reproductive research than in previous decades, there was still stigma associated with some reproductive topics, such as abortion, with several countries and organizations refusing to fund certain reproductive projects (Oudshoorn 2003). The USSR and the USA were also maneuvering geopolitically to gain world dominance, so creating a space for collaboration was seen to require “neutral” ground, in which many countries could come together without feeling threatened by their existing alliances (Confidential Letter 1968). Alongside geopolitical worries, the USA was seen to be a problematic country for contraceptive research due partly “to the litigious nature of medicine in the US” and intense Food and Drug Administration (FDA) regulations governing the testing of clinical substances (Conference 1959).

In 1968, Harkavy recommended the Karolinska Institute as the ideal location for a reproductive research center due to its “unparalleled prestige in scientific circles” (Confidential Letter 1968). The Karolinska Institute’s history of hosting renowned reproductive researchers was a significant consideration. This, along with Sweden’s historical support of family planning, was an important factor. In discussing the potential center with his Swedish counterparts at SIDA and the Karolinska Institute, Harkavy learned that the timing for establishing such a center was particularly good. In confidence, a prestigious Swedish researcher had relayed to Harkavy that the center’s proposal came “at the right psychological moment,” as a highly placed SIDA official believed “that SIDA has more money than it can usefully spend in support of overseas action programs (about $8 or 9 million in 1968–69) and is coming under criticism at home with regard to the amount of money SIDA is devoting to population work” (Confidential Letter 1968). In the late 1960s, SIDA was looking to invest in reproductive sciences, and diverting funding from population work was a possibility.

As early as 1966, SIDA’s working group on family planning questions had published a report recommending ongoing demographic and reproductive physiological research in order to successfully understand and implement birth control methods (Principal Points 1966). The report also outlined that, as IUDs and oral contraceptives were becoming more widespread in developing countries, Swedish research on such reproductive devices seemed particularly worthy of support. This, the report added, could potentially be more strategic than supplying aid to individual countries. In the report, the working group wrote:

It is remarkable that this research conducted in Sweden, which is internationally appreciated and plays an important role in the population problem, is almost exclusively funded by US funds. Some are driven almost exclusively by huge contributions made available by the National Institutes of Health, the Ford Foundation and the Population Council. However, it does not seem possible and reasonable to continue this research and education work over the long run without significantly increasing domestic support. (Principal Points 1966, 7)

While Sweden had hosted various reproductive researchers and reproductive projects, the funding had mostly been coming from international sources. The abortion law, research culture, and infrastructure for clinical trials supported research in Sweden, but foreign funds had also been integral to research success. SIDA’s interest in contributing financially to reproductive research was another indicator of the status of reproductive research in family planning and overpopulation circles.

The specific Swedish clinical trial infrastructure was also highly relevant to discussions of creating a reproductive research and training center. By the early 1970s, the WHO was also interested in the plans for a reproductive center, first initiated by SIDA, the Ford Foundation, and the Karolinska Institute. Alexander Kessler, head of the Human Reproduction Unit, WHO, reached out to Egon Diczfalusy, a renowned Hungarian-Swedish reproductive researcher working at the Karolinska Institute who had been part of earlier discussions of creating such a center (Kessler to Diczfalusy 1970). In late 1970, the Reproductive Endocrinology Research Unit at the Karolinska Institute became the host of the WHO Research and Training Centre on Human Reproduction, with Diczfalusy as the director (WHO 1970). While factors made the Karolinska Institute appealing, the Swedes had emphasized the specifics of the country’s abortion and clinical legal regulatory systems. In proposing an expansion of the Centre, the director of the Karolinska Institute and Diczfalusy discussed the merits of the Institute hosting the research center. They applauded the unique position the Institute and hospital were in, thanks to the country’s legal framework. Regarding reproductive research, they said: “The atmosphere for such research is particularly favorable in Sweden because of the law of interruption of gestation and the law regulating the clinical evaluation of new fertility controlling agents, including abortifacients” (Proposal 1971). Abortion pill research had driven the government to pass a proposition altering clinical testing conditions and making it possible to include pregnant women in trials. The particular benefits of allowing reproductive research and the development and use of birth control to help with overpopulation had also already been established with IUD testing. While the networks of researchers involved in IUD and abortion pill development were small, their impact was not. The specific arguments, clinical trials, as well as government and parliamentary discussions and decisions led to favorable reproductive research conditions. This, in turn, helped to secure an internationally lauded research and training center that was intended to host and train the next generation of scientists at an international level.

## Co-producing *Sweden* through reproductive research

By the late 1960s, Sweden was considered a strategic and useful partner in reproductive research networks. Influential individuals interested in family planning saw potential in the regulatory, legal, and scientific traditions of the country. At the same time, Sweden had been influenced by international interests and concerns, responding to the pressures to end global overpopulation by amending Swedish laws and policies. Various actors came together to co-produce ideas about Sweden that then made the country a valuable international partner.

Foreign aid actors had been working on bringing family planning into mainstream discussions for years. In 1951, Ernst Michanek, who would go on to be the director general of SIDA, had been cautioned not to bring up family planning at the United Nations Economic and Social Council, as any suggestion would simply be silenced procedurally (Population Crisis Hearings 1966). While changes had occurred by the early 1960s, there was still pessimism around the limited extent to which family planning was being supported by foreign aid. In a 1962 article, staff of the newly formed Council for International Development (NIB) was interviewed about birth control and foreign aid. *Science* reported:

Officials of the recently formed Swedish Agency for International Assistance are not overly optimistic about what can be done to decelerate population growth in the underdeveloped nations, but they realize that Sweden is the only aid-giving country that dares touch the subject. The Swedes, in determining where their relatively limited resources could be most effective in the underdeveloped countries have decided to emphasize birth control. (Population Crisis Hearings 1966, 560)

In these foreign aid networks, Sweden was cast as alone in its commitment to family planning as foreign aid, as exceptional and forward thinking. In 1966, SIDA sent three representatives to the USA to act as experts at a series of congressional Population Crisis Hearings (1966). These representatives were Michanek, then director general of SIDA, Ulf Borell, physician, professor of obstetrics and gynecology, and chair of SIDA’s advisory group on family planning, and Carl Wahren, deputy head of SIDA’s planning division. The three men traveled to Washington to advise the US Senate on Swedish family planning efforts both domestically and abroad. Seven months prior, the Swedish family counselor, teacher, and author, Birgitta Linnér, had given testimony about family planning programs to the same subcommittee (Population Crisis Hearings 1966). She had spoken briefly about research done by Borell at the Karolinska Institute and, when pressed for more details, recommended that the subcommittee reach out to the Swedish embassy to find out more. The visit of the three SIDA representatives was the result.

In their testimony, the representatives gave a broad overview of Sweden’s population issues, going back to being sparsely populated in the nineteenth century, and of the ideals of family planning—that every child should be wanted and that the masses should be educated properly in birth control techniques. They also presented research on human reproduction that was occurring in Sweden. In his address, Michanek said that, “Swedish physicians, in general, are not inhibited by ethical or religious reasons for prescribing contraceptives” (Population Crisis Hearings 1966, 556). In these discussions, Sweden appeared to be beyond ethical and religious worries around birth control, as well as experienced in providing family planning as foreign aid, invested in reproductive research, and interested primarily in these fields so that “every child should be wanted.” Michanek added that, “Swedish research in human reproduction is characterized by the philosophy that whenever possible, basic information on these processes should be obtained in clinical experimentation” (Population Crisis Hearings 1966, 558). In this regard, the men stressed that more research needed to be conducted in order to make the best birth control technologies possible. The American senate’s reaction to the testimony was positive. One of the senators made a point of exclaiming that Scandinavians were “very enlightened people” (Population Crisis Hearings 1966, 569).

In these instances, when Swedish actors traveled abroad, the presentation of Sweden is quite clear. Here, the representatives were required to explain and position Sweden to a foreign audience, highlighting how Sweden, through family planning and reproductive research, was being presented to the outside world. Sweden was not isolated from international concerns, and SIDA’s emphasis on family planning was also a result of larger discourses including other nations. As Alison Bashford has shown in her work, by the mid-1960s, family planning was becoming increasingly mainstream in international forums. In 1966, Burmese UN Secretary-General U Thant “made widely publicized declarations … that the international community must accord to parents the right to determine the numbers of their children” (Bashford 2013, 345). Sweden’s position on family planning and birth control was not as unique as it once was. As Haldor Byrkjeflot et al. (2022, 4) wrote, “The Nordic model was not the result of splendid Nordic isolation, but of national Nordic processes constantly interacting with the outside world.” While Sweden was one of the earliest nations to provide family planning as aid, the country was also continuously engaging with the international community in various forums such as the UN and reproductive research networks. Foreign interest in IUDs, for example, prompted Swedish researchers to engage with the technology on a local scale. Overpopulation concerns and family planning methodology were co-produced in complex networks of Swedish and non-Swedish actors.

However, these family planning actors in particular played an important role in co-producing reproductive research conditions in Sweden. The emphasis on overpopulation, starvation, and birth control was extremely important in discussions of what should and should not be allowed in Sweden. The inclusion of reproductive research and birth control development within family planning interests created real, infrastructural opportunities for reproductive researchers. The specifics of the clinical trials also helped to make conditions for infrastructure possible, as the clinical trials forced discussions that altered legal and policy guidelines for testing birth control technologies. Without testing IUDs and developing abortion pills, Swedish legal conditions would not have been as accommodating when SIDA, the Karolinska Institute, and the Ford Foundation began planning for a reproductive research center. Through these networks, Sweden was co-produced as neutral, rational, scientific, and legally conducive for clinical trials.

## Conclusion

In 1968, Sweden seemed the natural choice for a reproductive research site. Sweden had notable reproductive researchers, state investment in birth control development, and favorable legislation. American researchers feared that their own domestic circumstances would hamper clinical trials, and Sweden seemed like the answer to this issue. This paper has shown that Sweden’s international status in the late 1960s emerged thanks to a complex, tight-knit group of actors and that, while Sweden had a unique standing in terms of what the country could offer to reproductive research, this was also due to Sweden’s interaction and influence abroad. Without international worries about overpopulation, an interest in international aid, and consistent funding from abroad, the reproductive research circumstances in Sweden would not have been the same.

Domestic birth control development helped to situate Sweden internationally, profiling science and expertise as Sweden’s main contributions in development aid and portraying the country positively abroad. Specifically, developing and testing IUDs and abortion pills co-produced understandings of reproduction and shaped conditions for future reproductive research. In their work, Norén et al. (2023) argued that the smallness of Nordic countries contributed to the myth-making about the Nordic model. Small countries, they argued, “do not function simply as scaled-down large nations.… the dynamics of civil society and government, not to mention the circulation of people, material goods and media objects, function in particular ways in a small population” (Norén et al. 2023, 12). In this case, the local and small-scale qualities of the clinical trials, and how the actors involved in these trials were often also involved in legislating medical matters, co-produced favorable ideas of Sweden.

State involvement was also an important factor in many instances of birth control development, and actors were entangled in their various interests, coming together to create conducive conditions for reproductive research. Reproductive researchers, the National Board of Medicine, the Department of Justice, the Abortion Act, lawyers, the Swedish government, the Swedish parliament, RFSU, MHB, SIDA, the Ford Foundation, the WHO, and the Karolinska Institute were all actors in this network concerned with birth control and global overpopulation. This network of actors extended beyond Sweden to related networks, and following these networks shows how many actors were involved in making reproductive research possible.

This success in establishing a reproductive research center at the Karolinska Institute was also due in part to how Sweden was viewed—these networks of actors co-produced *Sweden* in various positive ways, and this played a role in promoting Sweden in international milieus. As this paper has argued, the technological development of birth control in Sweden helped create conducive conditions for international reproductive research collaboration, which secured Sweden’s international standing in development aid. This portrayal of Sweden as scientific, expert driven, and modern was not unique, and other scholarship has shown that these qualities were incorporated into versions of the Nordic model during this era. Foreign countries might have expected these qualities from Sweden. What this paper has presented, however, are specific instances in which Sweden was constructed as having these qualities and to what effect. It has demonstrated how clinical trials made international collaboration possible, the close connection between international aid and reproductive research in Sweden, and how decisions made in these networks were not always based on rational scientific processes. The ways in which Sweden was constructed in these networks had real practical and physical ramifications for scientific development and foreign aid efforts in the form of an international research hub.

Analyzing the micro level of local clinical trials and the macro level of larger global worries about overpopulation illustrates that, while prescient in their interest in family planning, foreign aid, and overpopulation, actors in Sweden were also deeply influenced by larger international concerns and were not isolated from other international actors. While Swedish historical contexts made certain opportunities for birth control development possible, they were neither predetermined nor the results of a singular nation acting above all others in a rational manner. Instead, the specifics of Swedish birth control development were closely entwined with larger international issues, even as they positioned Sweden as a unique nation for collaboration on reproductive research.

## Data Availability

The author confirms that the data supporting the findings of this study are available within the article (and/or) its supplementary materials.
